# Artificial intelligence: A transformative tool in precision oncology

**DOI:** 10.18632/oncotarget.28639

**Published:** 2024-08-26

**Authors:** Jeremy McGale, Matthew J. Liao, Egesta Lopci, Aurélien Marabelle, Laurent Dercle

**Keywords:** immunotherapy, oncology, artificial intelligence, radiomics, lactate dehydrogenase, PET, MRI, CT, SPECT

## Abstract

Artificial intelligence (AI) is revolutionizing society and healthcare, offering new possibilities for precision medicine. Immunotherapy in oncology (IO) has similarly transformed cancer treatment through novel mechanisms of therapeutic action, but has also led to atypical response patterns that challenge traditional methods for response evaluation. This editorial explores the role of AI in addressing these challenges through the development of new biomarkers for precise disease characterization, and in particular those built on imaging for the early response assessment of patients diagnosed with cancer and treated with IO. Properly leveraged AI-based techniques could herald a new era of precision medicine guided by non-invasive, imaging-based disease evaluation.

Immunotherapy in oncology (IO) has significantly improved outcomes for many cancers, leading to its increased use in various lines of therapy. This category of treatment includes immune checkpoint inhibitors, CAR-T cells, immunocytokines, as well as oncolytic viruses, all of which inherently harness a patient’s own immune system to more effectively target malignancy. However, IO often leads to atypical response patterns that are not effectively captured by conventional size-based imaging response criteria such as RECIST (Response Evaluation Criteria in Solid Tumors) [1]. These phenomena overall complicate the evaluation of treatment efficacy and can include pseudoprogression (an initial increase in tumor size followed by eventual response), mixed responses (some lesions shrink while others grow), hyperprogression (rapid tumor growth after treatment), and an abscopal effect (local treatment of one lesion causes regression of distant metastases). The new patterns of response and progression emphasize a need for more tailored monitoring and assessment methods to accurately gauge the effectiveness of IO [2].

To address this need for new IO response assessment tools, research is ongoing with the aim of developing noninvasive artificial intelligence (AI) biomarkers from clinical, biological, and imaging data. AI offers an in-depth approach to biomarker discovery by allowing for the processing of vast, heterogeneous datasets to identify variables most strongly correlated with specific clinical outcomes. These variables are then integrated into predictive algorithms for use with new patient data, as seen in Figure 1, allowing for the calculation of a probabilistic value for potential outcomes and generating an overall output that informs clinicians on the likelihood of a patient experiencing said outcome. For example, the model could predict the presence of malignant disease, expression of specific pathological phenotypes, or a patient’s five-year survival rate. While this tool does not offer a definitive evaluation of a particular patient’s disease state, it provides additional information to assist clinicians in making precise treatment decisions.

**Figure 1:**
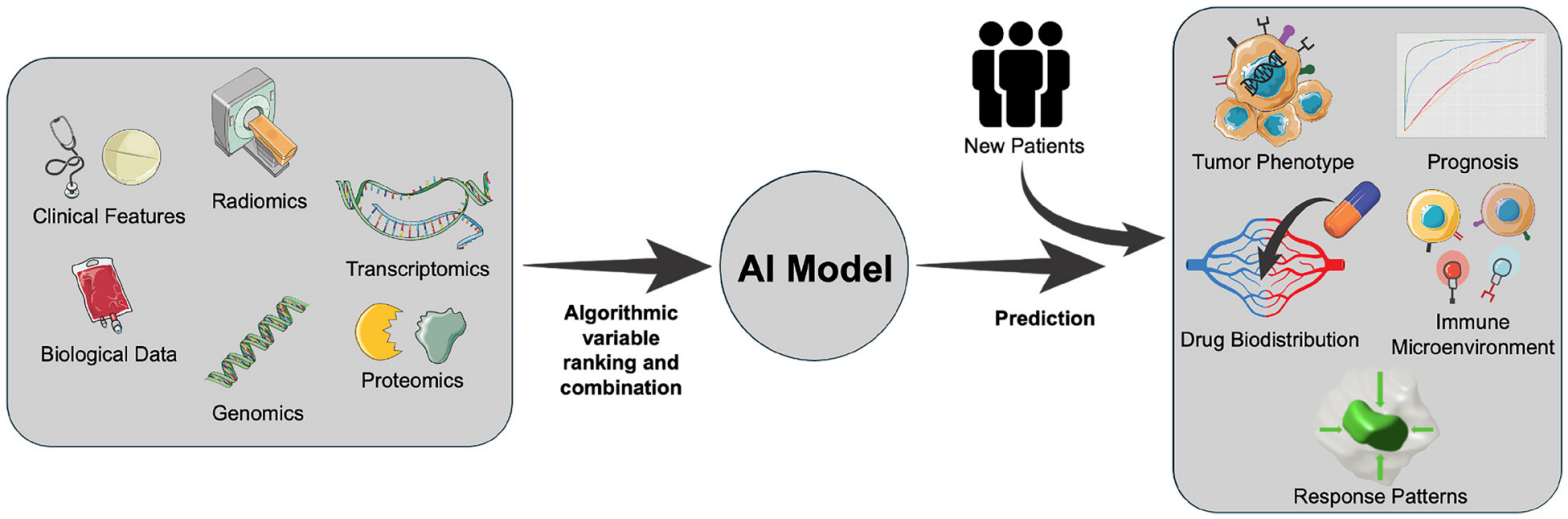
Overview of AI model development for use in oncology patients.

A recent study, titled “High serum LDH and liver metastases are the dominant predictors of primary cancer resistance to anti-PD(L)1 immunotherapy”, provides an example of utilizing AI to enhance patient care during IO administration [3]. 33 baseline variables were analyzed to determine correlation with overall survival (OS), with elevated baseline lactate dehydrogenase and the presence of liver metastases being identified as the most predictive factors. With this information, a model was developed to identify patients who were less likely to respond to IO and was found to be both prognostic and predictive. Patients with the above clinical features had significantly shorter OS and, interestingly, an elevated risk of hyperprogression, while those without were most likely to benefit long-term from IO.

Additionally, a comprehensive review of AI/radiomics applied to cross-sectional imaging (PET, CT, MRI) highlighted the current landscape in IO treatment [4]. Of 87 relevant studies, most utilized algorithms to predict treatment response or prognosticate survival at predetermined time points. However, these studies often had small training/testing cohorts (median of 101 patients), suggesting a risk of overfitting, and only two-thirds included discrete validation or testing datasets. The Radiomics Quality Score was used to assess each study and showed a median score of 12 out of a possible 36, indicating a need for more robust protocols, validation strategies, and stress testing to improve model generalizability. A subsequent study focusing on the role of AI/radiomics using PET and SPECT imaging in patients treated with IO demonstrated that most models have been created for a limited subset of tumor types and applications, and were mainly based on pretreatment radiomics features in lung cancer patients treated with anti-PD(L)1 IO [5]. Finally, a more generalized systematic review assessed AI-based methods for genomics, radiomics, digital pathology (pathomics), and clinical data analysis [6]. While the majority of included studies showed promise for AI-based prediction of IO response, none provided high-level evidence for immediate practice change, invoking a need for further prospective trials with direct validation of integration into clinical settings.

In conclusion, although AI/Radiomics in IO is a rapidly advancing field, there remains significant room for improvement. Primarily, developments such as non-invasive targeted radiotracers, e.g., radioisotopes synthesized with specific monoclonal antibodies such as those against CD8 or PD-(L)1, will enable more phenotype-specific imaging which may enhance AI performance [7]. Moreover, advanced techniques like magnetic resonance spectroscopy and hyperpolarized MRI will provide deeper insights into tumor biology, offering further data for AI model development [7]. Lastly, the quality of AI models will continue to improve as more rigorous standards for algorithm creation are implemented. These include stress testing, validation on large cohorts, and refined criteria for AI model appraisal. Although the application of AI and radiomics in the imaging of IO is still in an early phase, many advancements hold great promise for bridging the gap between experimental research and routine clinical practice.
